# Intracellular tortuosity underlies slow cAMP diffusion in adult ventricular myocytes

**DOI:** 10.1093/cvr/cvw080

**Published:** 2016-04-18

**Authors:** Mark Richards, Oliver Lomas, Kees Jalink, Kerrie L. Ford, Richard D. Vaughan-Jones, Konstantinos Lefkimmiatis, Pawel Swietach

**Affiliations:** 1 Burdon Sanderson Cardiac Science Centre, Department of Physiology, Anatomy and Genetics, Parks Road, Oxford OX1 3PT, UK; 2 Division of Cell Biology, Netherlands Cancer Institute, 1066 CX Amsterdam, Netherlands; 3 BHF Centre of Research Excellence, Oxford

**Keywords:** cAMP microdomains, Diffusion reaction, Mathematical modelling, Microfluidics, FRET sensor

## Abstract

**Aims:**

3′,5′-Cyclic adenosine monophosphate (cAMP) signals in the heart are often confined to concentration microdomains shaped by cAMP diffusion and enzymatic degradation. While the importance of phosphodiesterases (degradative enzymes) in sculpting cAMP microdomains is well established in cardiomyocytes, less is known about cAMP diffusivity (*D*_cAMP_) and factors affecting it. Many earlier studies have reported fast diffusivity, which argues against sharply defined microdomains.

**Methods and results:**

[cAMP] dynamics in the cytoplasm of adult rat ventricular myocytes were imaged using a fourth generation genetically encoded FRET-based sensor. The [cAMP]-response to the addition and removal of isoproterenol (β-adrenoceptor agonist) quantified the rates of cAMP synthesis and degradation. To obtain a read out of *D*_cAMP_, a stable [cAMP] gradient was generated using a microfluidic device which delivered agonist to one half of the myocyte only. After accounting for phosphodiesterase activity, *D*_cAMP_ was calculated to be 32 µm^2^/s; an order of magnitude lower than in water. Diffusivity was independent of the amount of cAMP produced. Saturating cAMP-binding sites with the analogue 6-Bnz-cAMP did not accelerate *D*_cAMP_, arguing against a role of buffering in restricting cAMP mobility. cAMP diffused at a comparable rate to chemically unrelated but similar sized molecules, arguing for a common physical cause of restricted diffusivity. Lower mitochondrial density and order in neonatal cardiac myocytes allowed for faster diffusion, demonstrating the importance of mitochondria as physical barriers to cAMP mobility.

**Conclusion:**

In adult cardiac myocytes, tortuosity due to physical barriers, notably mitochondria, restricts cAMP diffusion to levels that are more compatible with microdomain signalling.

## 1. Introduction

Signalling triggered by 3′,5′-cyclic adenosine monophosphate (cAMP) plays an essential role in cardiac physiology. The biological effects of cAMP depend on the amount produced and the subcellular distribution of the signal. In cardiac myocytes, spatio-temporal [cAMP] dynamics are determined by production by adenylyl cyclases (ACs), diffusion, buffering, and degradation by phosphodiesterases (PDEs).^1^ In principle, changes to any of these factors could alter the range and extent of target activation. However, the importance of diffusion in shaping cAMP signals in cardiac myocytes has only been marginally addressed because of difficulties in obtaining robust diffusivity measurements.

Signalling microdomains can be assembled on a framework of strategically placed synthetic, degradative, and target proteins.^2,3^ Tethering of protein kinase A (PKA), the main cAMP effector, to specific subcellular compartments is achieved by A-kinase anchoring proteins (AKAPs).^2^ Bringing PDEs in close proximity to other elements of the cascade helps to terminate the cAMP signal once it has had its downstream effect.^4,5^ Thus, the distribution and activity of PDE enzymes are important in sculpting cAMP microdomains to activate selected effectors, while protecting others.^6–10^ Several studies have linked aberrant distribution or activity of synthetic and degradative enzymes with cAMP signalling in disease.^11,12^

The degree to which PDE activity is able to compartmentalize cAMP signals depends strongly on cytoplasmic cAMP diffusivity (*D*_cAMP_). The three-dimensional spread of cAMP molecules from a point source can be described mathematically as follows:^13,14^

1 $$[\text{cAMP}]=\frac{J_{\mathrm{AC}}}{4\cdot \pi \cdot D_{\mathrm{cAMP}}\cdot r}\cdot \text{exp}\left(-r\cdot \sqrt{\frac{k_{\mathrm{PDE}}}{D_{\mathrm{cAMP}}}}\right)$$

where *k*_PDE_ is the rate constant of cAMP degradation by PDEs, *J*_AC_ is the rate of cAMP production by ACs, and *r* is the distance from the site of production. This equation demonstrates that cAMP microdomains become more spatially confined but attain a higher [cAMP] at the source as diffusivity *D*_cAMP_ decreases. The equation also shows that *D*_cAMP_ and *k*_PDE_ are independent variables that must be considered separately in models of microdomain signalling.^15^ Although high *k*_PDE_ limits the spread of cAMP molecules, even the most robust characterization of PDEs cannot predict the radial [cAMP] profile if *D*_cAMP_ is not measured.

In water,^16^ cAMP diffuses rapidly at 444 µm^2^/s, and mathematical simulations^6,17,18^ have demonstrated that such diffusivity precludes sarcomeric-level microdomain signalling, because the diffusive flux of cAMP would not be terminated by the finite degradative capacity of PDEs. A notable study on adult cardiac myocytes expressing a genetically encoded cAMP sensor measured cAMP velocity,^15^ and from this estimated *D*_cAMP_ to be 136 µm^2^/s. However, the relationship between velocity (units: µm/s) and diffusivity (units: µm^2^/s) is not straightforward, particularly when geometry and boundary conditions are not accounted for. Also, this study measured apparent cAMP diffusivity which lumps diffusive and reactive fluxes together and cannot distinguish between them. As illustrated in Eq. (1), [cAMP] measurements cannot derive true *D*_cAMP_ if *k*_PDE_ is not considered as part of the calculation. For example, rapid degradation by PDEs will make the spatial spread of cAMP appear slow, without a change in diffusivity. A study on neonatal cardiac myocytes^18^ calculated the diffusion of photolytically released cAMP to be 200 µm^2^/s, but it is unclear whether this estimate also applies to the adult myocyte. A recent report^19^ showed that a fluorescent cAMP derivative diffuses slowly in adult myocytes owing to a buffering effect, but it is unclear whether this also applies to endogenously produced cAMP at physiological concentrations. Measurements in other cell types have arrived at fast cAMP diffusivity (270,^20^ 330,^21^ and 500 µm^2^/s^22^). Thus, the general consensus^23^ is for fast *D*_cAMP_, yet this is difficult to reconcile with microdomain signalling. Accurate *D*_cAMP_ measurements in adult cardiac myocytes are warranted for more quantitative understanding of the limits of local signalling.

We measured endogenously generated cAMP by confocally imaging rat ventricular cardiomyocytes expressing an adenovirally delivered FRET (Förster resonance energy transfer)-based sensor reporting [cAMP] in cytoplasm.^24^ Compared with other sensors used previously in adult cardiac myocytes, H187 offers a substantially improved dynamic range for imaging [cAMP]. PDE activity (*k*_PDE_) was measured on a cell-by-cell basis to obtain reaction fluxes necessary for solving the diffusion reaction equation that describes [cAMP] dynamics. To measure diffusivity, [cAMP] gradients were generated in cytoplasm by exposing one half of a myocyte to agonist using a dual microperfusion apparatus,^25,26^ similar to a technique used previously to study the effects of [cAMP] non-uniformity on Ca^2+^ current.^27^ The size of the [cAMP] gradient between the net-producing (agonist-exposed) and net-consuming (antagonist-exposed) ends of the cell provides a read out of *D*_cAMP_, calculated to be 32.3 ± 7.6 µm^2^/s. We show that low *D*_cAMP_ is not a consequence of cAMP buffering, but arises from the tortuosity imposed by physical barriers inherent to cardiac myocyte ultrastructure,^28^ particularly mitochondria. Our measurement of *D*_cAMP_ in adult cardiac myocytes is more compatible with microdomain signalling delimited by PDE activity.

## 2. Methods

### 2.1 Isolation and viral transduction of myocytes

Ventricular myocytes were isolated from male Sprague Dawley rats (300–325 g) using enzymatic digestion and mechanical dispersion. Animals were sacrificed by stunning followed by cervical dislocation in accordance with UK Home Office regulations (Schedule I of A(SP)A 1986), approved by national and University ethics committees. See Supplementary material online, *Methods*. Cells were either used for experiments on the same day or cultured overnight for experiments with H187. Myocytes were cultured overnight on µ-slides (Ibidi, Germany) in myocyte culture medium (MEM supplemented with 9 mM NaHCO_3_, 1% l-glutamine, 1% penicillin/streptomycin), 0.5 µM cytochalasin D to preserve cell shape,^29^ and adenovirus containing Ad-EpacH187 cAMP FRET sensor construct (final titer 5 × 10^−9^ VP/mL). After overnight culture, the virus-containing medium was replaced with myocyte culture medium supplemented with 2.5% FBS. H187 fluorescence was observed at 24–30 h post-infection. Details of imaging and analyses are given in the Supplementary material online.

### 2.2 Solutions

Solutions were delivered at 37°C. Normal Tyrode contained (in mM) 135 NaCl, 4.5 KCl, 1 CaCl_2_, 1 MgCl_2_, 11 glucose, and 20 HEPES, at pH 7.4. Acetate-containing solutions had iso-osmotically replaced NaCl for NaAcetate. Na^+^-free, Ca^2+^-free solution contained 140 mM *N*-methyl-d-glucamine in place of NaCl and 1 mM EGTA in place of CaCl_2_.

### 2.3 Statistics

Differences were tested by *t*-test at 5% significance. Data are reported as mean ± SEM (number of cells/number of animals).

## 3. Results

### 3.1 Calibrating the cAMP sensor

The genetically encoded FRET-based cAMP sensor H187 offers a superior dynamic range for monitoring cAMP,^30^ necessary for resolving *D*_cAMP_. H187 was excited by an argon laser, and FRET was reported as the ratio of fluorescence measured at 480 ± 10 and 530 ± 10 nm (see Supplementary material online, *Figure S1A*). Expression of H187 and overnight culture in the presence of cytochalasin D did not detubulate cardiac myocytes, nor affect mitochondrial density (see Supplementary material online, *Figure S1B*). To calibrate FRET ratio into units of [cAMP], H187 protein was obtained from HEK293 cells expressing the sensor. Cells were lysed in internal buffer solution titrated to pH 7.2 or 6.6, containing a range of [cAMP] and the PDE inhibitor IBMX (100 µM) to prevent cAMP degradation. Supplementary material online, *Figure S2* shows the calibration curves fitted to the Grynkiewicz equation describing minimum and maximum ratio (*R*_min_, *R*_max_) and apparent affinity (*K*_cAMP_). Low pH (6.6) did not affect *K*_cAMP_ (11.14 µM at pH 7.2 and 11.48 µM at pH 6.6) but increased *R*_min_ (from 0.6727 to 0.7531) and *R*_max_ (from 1.2386 to 1.313).

### 3.2 Generating solute gradients using dual microperfusion

**Figure 1:**
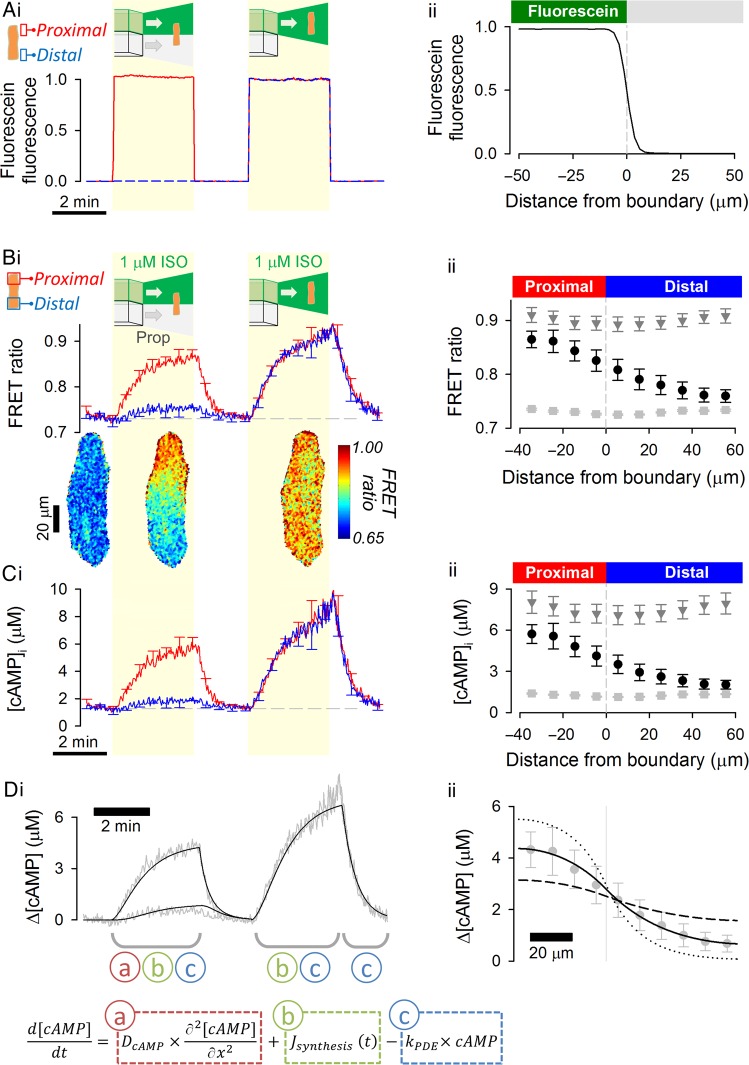
cAMP diffusivity measured using the H187 sensor is low in the cytoplasm of adult ventricular myocytes. (*A*) The experimental protocol is visualized with fluorescein added to one of two microstreams. (i) Time course of fluorescence emitted from extracellular fluorescein surrounding the proximal and distal regions of interest (ROI) of a myocyte. Manoeuvres 1 (both microstreams are released) and 2 (only fluorescein-containing microstream is released) are shaded yellow. (ii) Profile of fluorescence across the boundary between microstreams, showing the sharp (<10 µm) degree of microstream separation (*n* = 10/3). (*B*) Experiment on H187-expressing myocyte. 1 μM isoproterenol (ISO) included in one microstream (green) to active β-adrenoceptors; other microstream contained 10 µM propranolol, β-adrenoceptor antagonist (*n* = 11/4). (i) Time course of FRET ratio in proximal and distal ROIs; (ii) Longitudinal profile of FRET ratio, relative to boundary position, measured under resting conditions (light grey squares), during the last 10 s of Manoeuvre 1 (black circles) and during the last 10 s of Manoeuvre 2 (dark grey triangles). (*C*) Calibrated [cAMP] (i) time course and (ii) longitudinal profiles. (*D*) Mathematical model fit to experimental data. Best-fit *D*_cAMP_ = 35 ± 3.4 µm^2^/s indicates slow cAMP diffusion inside adult cardiac cytoplasm. To illustrate the goodness-of-fit to data, longitudinal profiles were also simulated for a four-fold lower (dotted lines) and a four-fold higher (dashed lines) *D*_cAMP_. Inset: the partial differential equation describing cAMP dynamics is a linear combination of three processes (labelled a, b, and c). These can be parameterized sequentially by analysing different stages of the experiment. Dynamics in the third stage are determined by degradation only (c). With this information, it is then possible to characterize synthesis (b) by analysing the second stage. Having parameterized b and c, diffusion (a) can be obtained by analysing the first stage.

A square-bore, double barrel microperfusion device, shown in Supplementary material online, *Figure S3*, was used to release two sharply separated microstreams.^25,26^ When the inter-stream boundary is positioned perpendicular to a myocyte's long-axis, the two ends of the cell are exposed to different solutions. The high degree separation between microstreams was tested by including fluorescein (30 µM) in one microstream only (see Supplementary material online, *Figure S3C*; *Figure 1A*). The same cell can then be exposed uniformly to one microstream only by closing the flow of the other microstream (see Supplementary material online, *Figure S3D*; second part of the protocol in *Figure 1A*i). The time constant of solution exchange is exceptionally fast (20 ms).^31^

### 3.3 Calculating cAMP diffusivity from evoked [cAMP] gradients

The experimental protocol for studying cAMP dynamics consisted of two manoeuvres. In the first manoeuvre, dual microperfusion delivered a β-agonist (1 µM isoproterenol; ISO) to one end of the cell and a β-antagonist (10 µM propranolol; Prop) to the other half. In the second manoeuvre, the myocyte was exposed uniformly to ISO. Under resting conditions and after each manoeuvre, the myocyte was uniformly exposed to propranolol. *Figure 1B*i shows time courses of FRET ratio in proximal and distal regions of the cell, defined as measurement areas at opposite poles of the cell, equal to one-quarter of the myocyte length. FRET ratios were converted to [cAMP] (*Figure 1C*i) using calibration curves (see Supplementary material online, *Figure S2*). Longitudinal FRET ratio and [cAMP] profiles under resting conditions and during the last 10 s of the first and second manoeuvre are plotted in *Figure 1B*ii (FRET ratio) and *Figure 1C*ii ([cAMP]).

During the first manoeuvre (*Figure 1C*), any rise in [cAMP] in the propranolol-exposed half of the cell must be explained by diffusion from the ISO-exposed region. This experiment produces a state in which the ISO-exposed half of the cell is a net producer of cAMP (referred to as the proximal end), whereas the other half is a net consumer of cAMP (referred to as the distal end). The two domains are coupled by diffusion, resulting in a longitudinal [cAMP] gradient that is strongly dependent on *D*_cAMP_ (*Figure 1B*ii). Since the system approaches a steady state, fluorescence images can be time-averaged to improve the signal-to-noise ratio (*Figure 1B* insets). To calculate *D*_cAMP_ from the longitudinal [cAMP] gradient, the chemical reactions that influence [cAMP] dynamics must be quantified. Reaction kinetics were derived from [cAMP] dynamics during uniform exposure to ISO (the second manoeuvre) and subsequent ISO withdrawal (NB: for spatially uniform [cAMP] changes, net diffusive flux is zero). The rate of [cAMP] recovery upon ISO withdrawal provides a measure of the cAMP degradative capacity (i.e. PDE activity), modelled here as a first-order reaction (rate = *k*_PDE_ × [cAMP]). Supplementary material online, *Table S1* summarizes the results of fitting. The rate of cAMP synthesis, modelled as a time-dependent function (see Supplementary material online, *Table S1* legend), was inferred from the rate of [cAMP]-rise minus the calculated degradative flux (*k*_PDE_ × [cAMP]). Thus, a single experiment provides a dataset for quantifying cAMP degradation, synthesis, and diffusion. The mathematical framework used to analyse these data is illustrated in *Figure 1D*. By least-squares best fitting to the experimental data, *D*_cAMP_ was measured to be 35 ± 3.4 µm^2^/s (see Supplementary material online, *Figure S3E*, compares the [cAMP] profile with the extracellular drug compartmentalization). Also shown in *Figure 1D*ii are simulations for four-fold higher and four-fold lower *D*_cAMP_ to demonstrate the sensitivity of the measured [cAMP] gradient to *D*_cAMP_. Overall, these results show that cAMP diffusivity in cardiac cytoplasm is much slower than in water.

### 3.4 H187 does not interfere with the estimate of cAMP diffusivity

H187 could, in principle, interfere with normal cAMP diffusion. If, for instance, cytoplasmic mobility of H187 were very fast, the sensor could facilitate cAMP diffusion (i.e. overestimate *D*_cAMP_). If, on the other hand, H187 diffusion were negligible, the sensor could reduce *D*_cAMP_ by acting as a fixed buffer.

**Figure 2:**
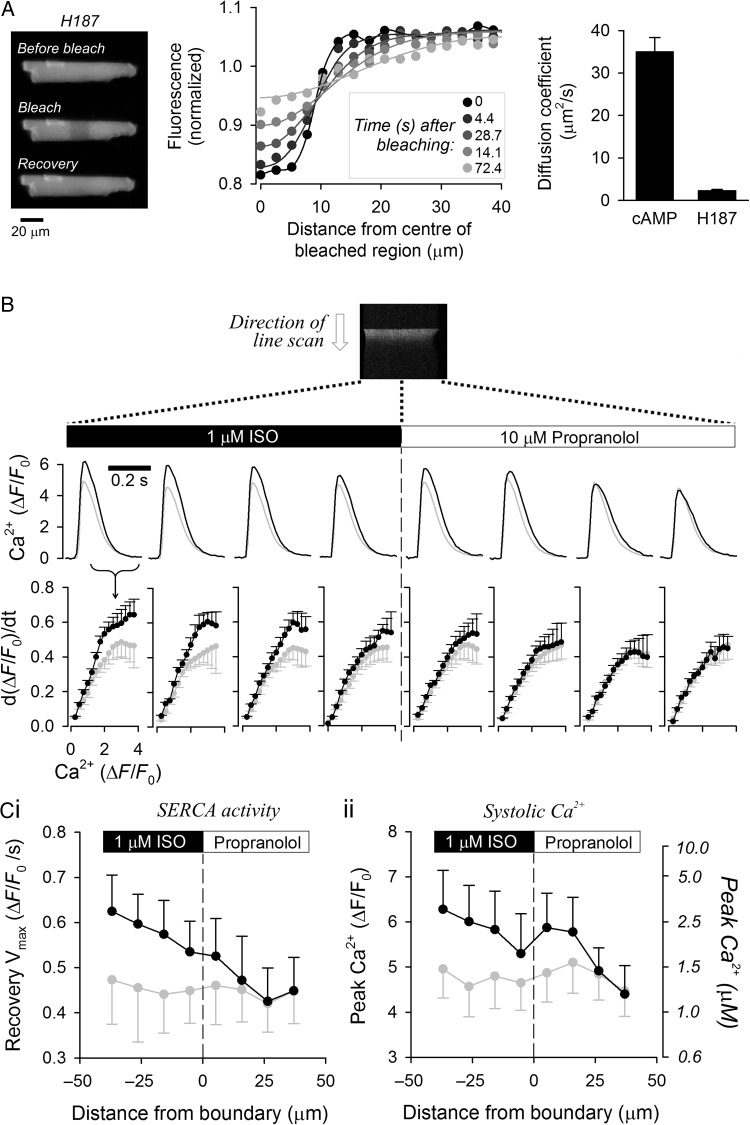
H187 does not interfere with the rate of cAMP diffusion. (*A*) Fluorescence recovery after photobleaching (FRAP) protocol for measuring H187 diffusivity in adult cardiac myocytes. Bleaching was performed in a 20 µm-wide region in the middle of the cell. Spatial profiles show fluorescence as a function of distance from the bleaching region at different time points during recovery. Continuous lines show best fit by diffusion model. H187 diffusivity (*n* = 17/3) was 15-fold slower than *D*_cAMP_ measured in *Figure 1*; therefore, H187 cannot meaningfully accelerate cAMP diffusion. (*B*) Using SERCA activity as a bioassay of [cAMP] in adult cardiac myocytes that are not expressing H187. Cell paced at 2 Hz and imaged for [Ca^2+^] with Fluo3 fluorescence in line scan mode. Ca^2+^ transients (CaTs) measured in line scan sections on either side of boundary between ISO-containing and propranolol-containing microstreams. (*B*) Rate of CaT recovery as a function of [Ca^2+^] provides a measure of SERCA activity before (grey) and during (black) dual microperfusion (*n* = 9/2). Activation of flux indicates that cAMP had activated SERCA pumps locally. (*C*) (i) Peak recovery of CaT and (ii) systolic [Ca^2+^] measured before (grey) and during (black) dual microperfusion (*n* = 9/2). Right axis shows calibrated units, with Fluo3 *K*_d_ = 840 nM. The longitudinal profiles of SERCA activity (in the absence of H187 sensor) are comparable to longitudinal [cAMP] profiles reported with H187 (see *Figure 1D*ii), indicating that H187 does not slow cAMP diffusion.

H187 diffusivity was measured in myocytes (superfused in Na^+^-free, Ca^2+^-free solution to block contraction) by observing the rate of diffusive dissipation of fluorescence (excited at 488 nm) following 25% signal photobleaching in a 20 µm-wide region at the middle of the myocyte (FRAP: fluorescence recovery after photobleaching). H187 fluorescence along the cell's major axis was normalized to the starting level and fitted with a diffusion model that assumes post-bleaching conservation of total fluorescence (*Figure 2A*). H187 diffusivity was measured to be 2.35 ± 0.25 µm^2^/s, i.e. 15-fold lower than *D*_cAMP_ (*P* < 10^−4^), indicating that the sensor (mol. wt ∼167 kDa) cannot meaningfully facilitate cAMP movement.

Due to its low diffusivity, H187 behaves as a fixed cAMP buffer. If the degree of buffering by H187 were substantial, then the [cAMP] response to ISO reported by FRET in H187-expressing cells (*Figure 1C*) would be more spatially restricted than in non-infected cells. To test this, the size of the [cAMP] gradient evoked by regional ISO exposure was inferred from the activity of the sarcoplasmic reticulum (SR) Ca^2+^ ATPase (SERCA) in wild-type myocytes. Electrical stimulation (2 Hz) triggered Ca^2+^ transients (CaTs) which were measured by Fluo3 fluorescence in line scan (*Figure 2B*). The recovery phase of CaTs measured in different regions of the cell provided a read-out of SERCA activity locally within regions of interest (NB: ROI positions were re-scaled during contractions). As expected, SERCA activity was stimulated in the ISO-exposed half of the cell. A modest increase in SERCA activity was also observed in the propranolol-exposed region, confirming the diffusive spread of cAMP. The longitudinal profile of SERCA activity (*Figure 2C*i) and peak systolic [Ca^2+^] (*Figure 2C*ii; related to SR load and hence SERCA activity) measured in wild-type myocytes were in agreement with the degree of cAMP diffusion reported with H187 in transduced cells (*Figure 1*). These results indicate that the level of additional cAMP buffering introduced into myocytes by H187 expression is small and does not significantly reduce cAMP diffusion.

### 3.5 Measuring cAMP diffusivity over a wider range of [cAMP]

**Figure 3:**
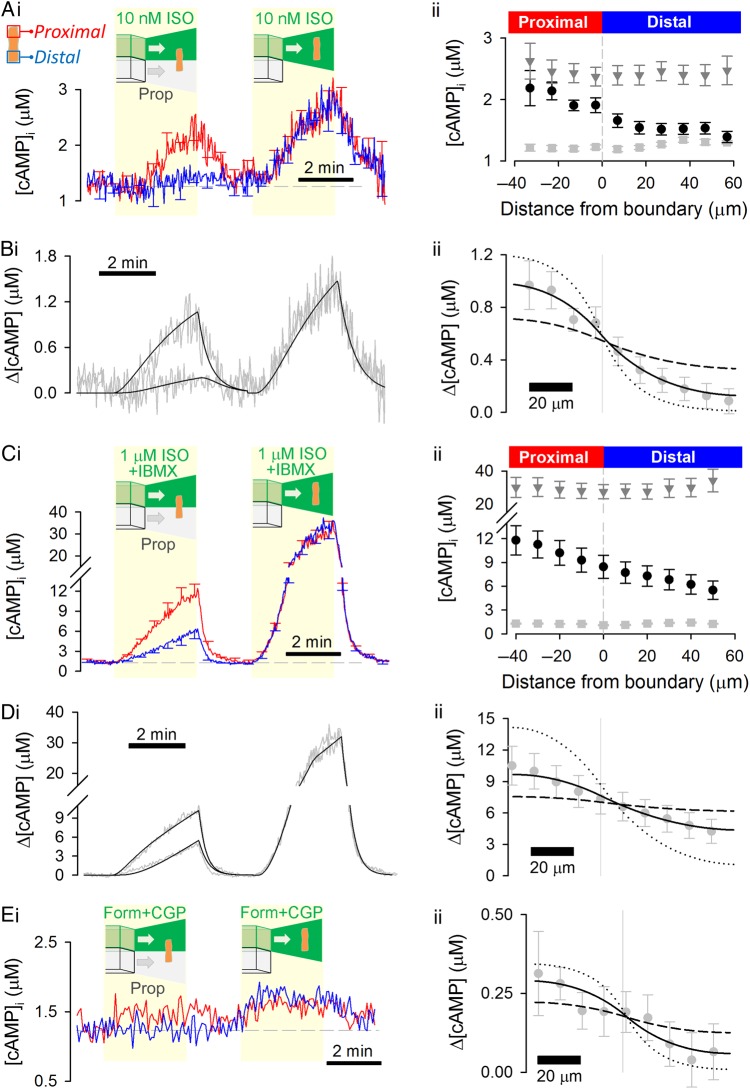
Cytoplasmic cAMP diffusivity is low, irrespective of the amount of cAMP produced or the type of β-receptor activated. (*A*) Experiment similar to that in *Figure 1B* repeated for lower (10 nM) dose of ISO (*n* = 15/4) to evoke a smaller proximal [cAMP]-rise. (*B*) Model fit to experimental data, with best-fit *D*_cAMP_ = 32 ± 8.7 µm^2^/s confirming slow diffusion. (*C*) Experiment similar to that shown in *Figure 1B* repeated with phosphodiesterase inhibitor IBMX (100 µM) present in microstream containing 1 µM ISO (*n* = 12/4) to evoke a larger proximal [cAMP]-rise. (*D*) Mathematical model fit to experimental data, with best-fit *D*_cAMP_ = 28 ± 3.0 µm^2^/s confirming slow diffusion. (*D*) Experiment similar to that in *Figure 1B* repeated using formoterol (100 nM) and CGP 20712A (1 µM) to selectively activate β_2_-receptors in the proximal end of the cell (*n* = 9/3). (i) The [cAMP]-rise was small, compared with the ISO response. A steady-state [cAMP] gradient can be resolved by time averaging over a 2-min interval during regional β_2_-adrenoceptor activation. (ii) Model fit to experimental data, with best-fit *D*_cAMP_ = 24 ± 15.8 µm^2^/s confirming slow diffusivity. To illustrate the goodness-of-fit data, longitudinal profiles in *B*ii, *D*ii, and *E*ii were also simulated for a four-fold lower (dotted lines) and a four-fold higher (dashed lines) *D*_cAMP_.

Measurements of *D*_cAMP_ were repeated with a lower dose of ISO (10 nM) that elicited a smaller [cAMP]-rise (*Figure 3A*). Again, a similar *D*_cAMP_ was measured (32 ± 8.7 µm^2^/s; *Figure 3B*; *P*= 0.78 vs. 1 µM ISO). Increasing the net production of cAMP at the proximal end of the cell by including the PDE inhibitor IBMX (100 µM) in the 1 µM ISO-containing microstream resulted in a larger [cAMP]-rise (*Figure 3C*). *D*_cAMP_ was 28 ± 2.9 µm^2^/s (*Figure 3D*) and not significantly different from the measurement obtained with 1 µM ISO (*P*= 0.13). Overall, these results indicate that *D*_cAMP_ is independent of the amount of cAMP produced, at least over the range reported by H187.

### 3.6 Measuring cAMP diffusivity evoked by β_2_-receptor activation

ISO acts on β_1_ and β_2_ receptors present in adult cardiac myocytes, but the majority of the [cAMP] response is attributable to the activation of β_1_ receptors.^15^ To test whether β_2_-evoked cAMP diffuses at a different rate to ISO-evoked cAMP, one half of a myocyte was exposed to the β_2_ agonist formoterol (100 nM) plus the β_1_ antagonist CGP 20712A (1 µM) to selectively activate β_2_ receptors (the distal end of the myocyte was exposed to 10 µM propranolol). The proximal [cAMP]-rise was small (*Figure 3E*i), consistent with previous observations,^15^ which necessitated a modified analytical approach. Time averaging over a 2-min period during dual microperfusion produced a smooth [cAMP] gradient for analysis. Since it was not possible to obtain an accurate measure of PDE activity because of the small [cAMP]-rise, *k*_PDE_ determined with ISO was used instead. The synthesis rate was fitted to a constant flux. This analysis yielded low *D*_cAMP_ (24 ± 15.8 µm^2^/s; *Figure 3E*ii) that was not significantly different from the diffusivity measured with 1 µM ISO (*P*= 0.09), indicating that cAMP produced in response to β_2_-selective or mixed β agonists is released into compartments that impose similar diffusive restrictions.

### 3.7 Measuring cAMP diffusivity in acidified cytoplasm

**Figure 4:**
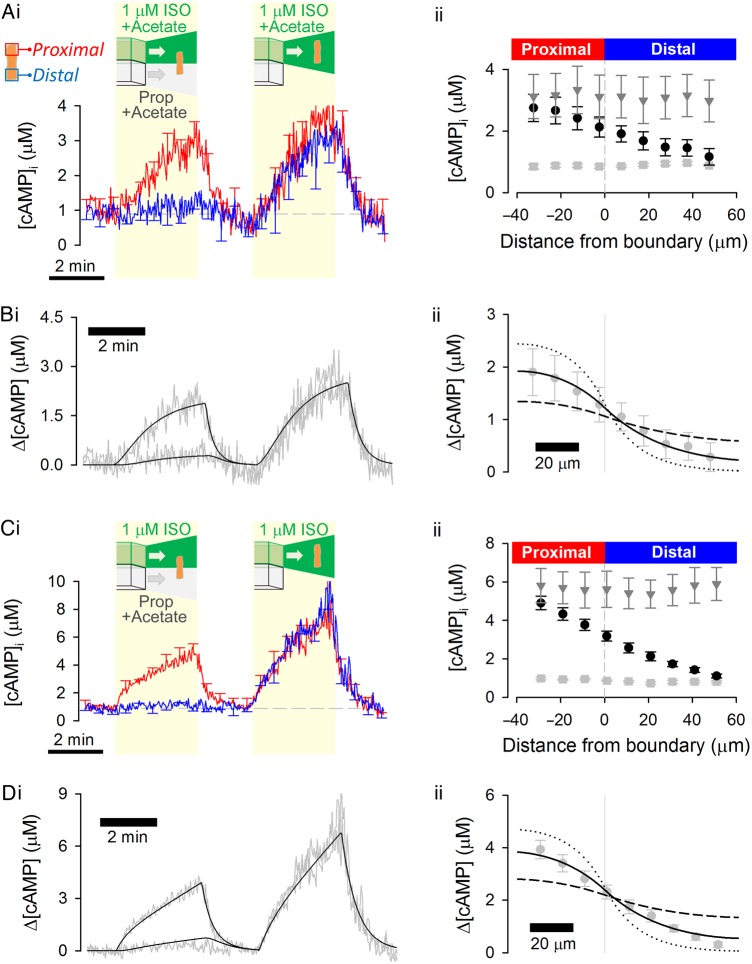
Cytoplasmic cAMP diffusivity is not changed by cytoplasmic acidification. (*A*) Experiment similar to that shown in *Figure 1B* repeated on myocytes exposed to 80 mM acetate to acidify cytoplasm uniformly (*n* = 7/3). 5-(*N,N*)-dimethylamiloride (DMA; 30 µM) was included in both microstreams to inhibit pH regulation by Na^+^/H^+^ exchanger 1. (*B*) Model fit to experimental data, with best-fit *D*_cAMP_ = 41 ± 12.2 µm^2^/s confirming slow diffusivity. (*C*) Experiment shown in *Figure 4A* repeated, but with acetate in the distal microstream only; DMA (30 µM) was included in both microstreams (*n* = 6/3). This protocol produces a pH-gradient overlying the gradient of β-adrenoceptor activation; higher pH in ISO-containing microstream favours greater cAMP production. (*D*) Model fit to experimental data with best-fit *D*_cAMP_ = 32 ± 4.8 µm^2^/s confirming slow diffusivity. To illustrate the goodness-of-fit data, longitudinal profiles in *B*ii and *D*ii were also simulated for a four-fold lower (dotted lines) and a four-fold higher (dashed lines) *D*_cAMP_.

The electric charge on cAMP (−1) and interacting proteins could influence *D*_cAMP_. This was explored by increasing the overall protonation state. Cytoplasm was acidified to pH 6.6 by uniform exposure to 80 mM acetate. 5-(*N*,*N*)-dimethylamiloride (DMA; 30 μM) was included to stabilize the acid load by inhibiting Na^+^/H^+^ exchanger-1 (NHE1) (*Figure 4A*).^26^ The H187 calibration measured at pH 6.6 (see Supplementary material online, *Figure S2B*) was applied to convert FRET ratio into [cAMP]. The amount of cAMP produced with 1 µM ISO is reduced at acidic pH, but *D*_cAMP_ (41 ± 12.2 µm^2^/s; *Figure 4B*) was not significantly different from that measured at physiological pH (7.2; *P*= 0.57). To evoke a larger [cAMP]-rise and observe its spread into an acidified cytoplasmic compartment, a gradient of intracellular pH was imposed by including 80 mM acetate in the distal microstream only (both microstreams contained 30 µM DMA to stabilize the ensuing pH gradient; *Figure 4C*).^26^ Under this protocol, the ISO-exposed half of the cell is at physiological pH, while the opposite end is acidic. FRET ratio was converted to [cAMP] by interpolating H187 calibration curves between pH 7.2 and 6.6 and using previous measurements of pH under a similar protocol^26^ (see Supplementary material online, *Figure S2*). Again, *D*_cAMP_ (32 ± 4.8 µm^2^/s; *Figure 4D*) was not different from measurements made at physiological pH, arguing that protonation state does not affect *D*_cAMP_ (*P*= 0.61 vs. pH 7.2).

### 3.8 Measuring cAMP diffusivity in cytoplasm saturated with a cAMP analogue

**Figure 5:**
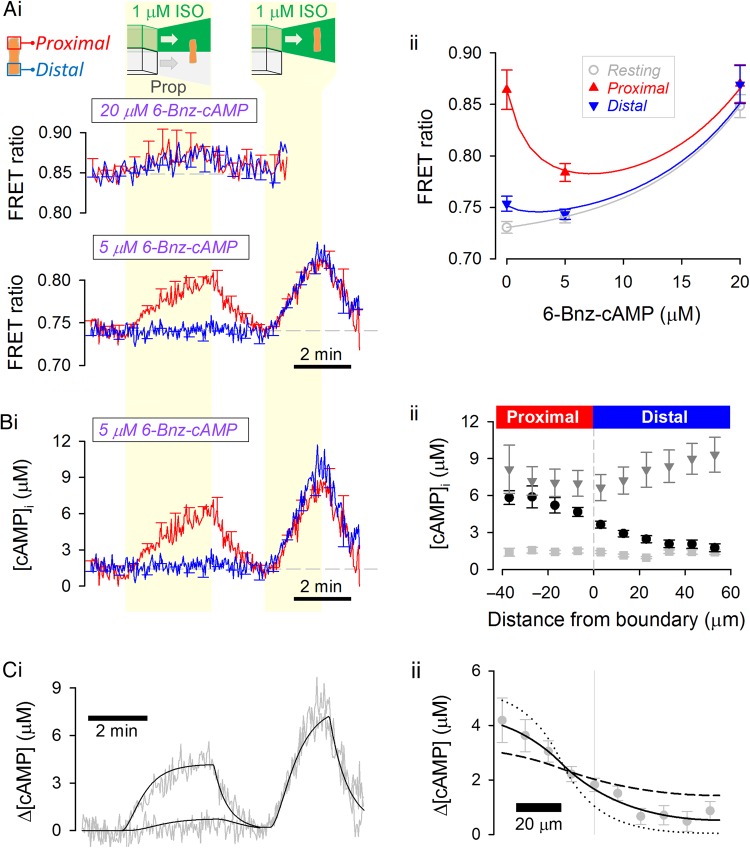
Cytoplasmic cAMP diffusivity is not affected by saturating cytoplasmic cAMP-binding sites with the analogue 6-Bnz-cAMP. (*A*) (i) Experiment similar to that shown in *Figure 1B* performed on myocyte AM-loaded with 20 µM (upper panel; *n* = 7/3; measured at half the normal acquisition frequency) or 5 µM (lower panel; *n* = 11/3) 6-Bnz-cAMP, a cAMP analogue with affinity for cAMP-binding sites. (ii) FRET ratio under resting conditions and at the proximal and distal end of the cell during dual microperfusion. The decreasing magnitude of FRET response to proximally presented ISO with higher doses of 6-Bnz-cAMP is due to a right shift in the [cAMP]-H187 calibration curve. (*B*) (i) [cAMP] time course in cells loaded with 5 µM 6-Bnz-cAMP. (ii) Longitudinal [cAMP] profiles, relative to boundary position, measured under resting conditions (light grey squares), during the last 10 s of dual microperfusion (black circles) and during the last 10 s of uniform exposure to agonist (dark grey triangles). (*C*) Model fit to (i) time course and (ii) longitudinal profile. Best-fit *D*_cAMP_ = 29 ± 7.1 µm^2^/s is not different from measurements made in the absence of 6-Bnz-cAMP. To illustrate the goodness-of-fit data, longitudinal profiles were also simulated for a four-fold lower (dotted lines) and a four-fold higher (dashed lines) *D*_cAMP_.

Diffusivity of cAMP may be reduced by buffering to proteins, such as the regulatory subunits of PKA.^32,33^ The effect of these buffering sites on *D*_cAMP_ was explored by introducing a cAMP analogue that saturates binding sites. 6-Bnz-cAMP (N^6^-Benzoyladenosine-cAMP) was chosen as a suitable analogue, because it avidly binds and activates PKA, but does not activate EPAC^34^ on which H187 is based.^30^ Although 6-Bnz-cAMP has no meaningful efficacy on EPAC sites, it nonetheless will complete, to some degree, with cAMP for binding to H187. This ‘competitive antagonism’ would shift the [cAMP]-H187 calibration towards higher [cAMP]. To quantify this shift, H187-expressing adult myocytes were AM-loaded with 6-Bnz-cAMP and regionally exposed to ISO (*Figure 5A*). When 20 µM of the analogue was used, the FRET ratio was essentially insensitive to 1 µM ISO, indicating that at this dose, the analogue shifts the sensor's cAMP affinity out of the physiological range. In myocytes loaded with 5 µM 6-Bnz-cAMP, the FRET response to ISO was measureable, and this dose of analogue was chosen as a compromise between adequate saturation of PKA sites (0.5–1 µM)^6^ and acceptable H187 resolving power.

To characterize how 6-Bnz-cAMP affects the H187 calibration curve, the sensor was expressed in rat neonatal myocytes (see Supplementary material online, *Figure S4*). Addition of the AM-ester of 6-Bnz-cAMP (5 µM) did not affect resting FRET ratio (see Supplementary material online, *Figure S4C*i), nor the FRET ratio change upon treatment with forskolin (10 µM) plus IBMX (100 µM) (see Supplementary material online, *Figure S4C*ii) which raises intracellular [cAMP] to levels in excess of [6-Bnz-cAMP]. These observations indicate that 6-Bnz-cAMP does not affect *R*_min_ and *R*_max_ of the calibration curve. ISO (10 nM) evoked a FRET response that was smaller in 6-Bnz-cAMP pre-loaded cells compared with control myocytes (see Supplementary material online, *Figure S4*). This confirms that 6-Bnz-cAMP competes with cAMP for binding to H187 and decreases the sensor's apparent cAMP affinity, in agreement with published binding constants.^34^

Knowing that 6-Bnz-cAMP does not affect *R*_min_ and *R*_max_, it is possible to estimate the factor by which *K*_cAMP_ is shifted by comparing the FRET response to 1 µM ISO in the presence and absence of the analogue (*Figure 5A*ii). 6-Bnz-cAMP-AM (5 μM) increased *K*_cAMP_ in adult myocytes by a factor of 3.02. This relatively modest right-shift in the calibration curve does not impair the sensor's ability to resolve [cAMP] gradients evoked by ISO. [cAMP] time courses and longitudinal profiles were thus generated using this shifted calibration curve (*Figure 5B*). *D*_cAMP_ measured from the [cAMP] response to regional exposure to 1 µM ISO (29 ± 7.1 µm^2^/s; *Figure 5C*) was the same as in myocytes that had not been loaded with 6-Bnz-cAMP (*P*= 0.45). In summary, saturating cAMP-binding sites with a cAMP analogue does not accelerate *D*_cAMP_, indicating that cAMP buffering is not normally a substantial restriction to cytoplasmic cAMP mobility.

### 3.9 cAMP diffusivity is independent of the concentration of cAMP produced

**Figure 6:**
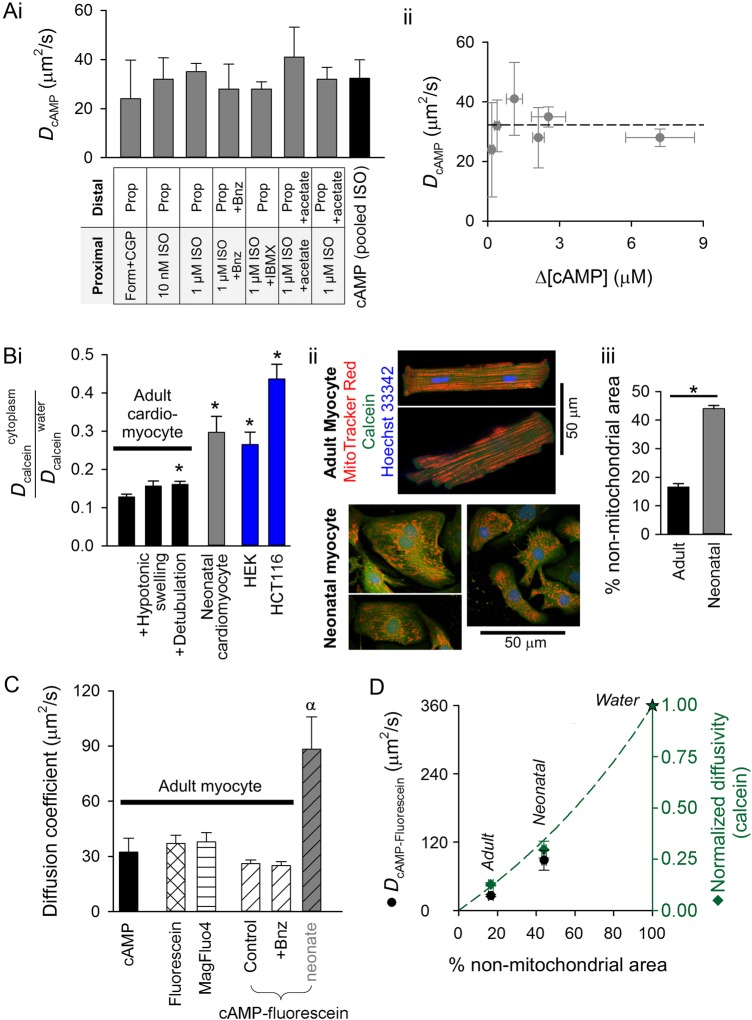
Cytoplasmic cAMP diffusivity in adult myocytes is comparable to that of similar sized molecules and is restricted by tortuosity. (*A*) (i) Summary of *D*_cAMP_ measurements made by imposing [cAMP] gradients in adult myocytes by regional exposure to agonist. (ii) *D*_cAMP_ is independent of the mean [cAMP]-rise evoked by regional exposure to agonist. (*B*) (i) Probing cytoplasmic tortuosity in terms of cytoplasmic calcein diffusion (measured by fluorescence recovery after photobleaching protocol) relative to calcein diffusivity in water (604 µm^2^/s). Measurements in adult cardiac myocytes under control conditions (*n* = 16/3), following hypotonic swelling (200 mOsm/kg solution; *n* = 14/3) or following detubulation with formamide (*n* = 14/3), and in HEK293 cells (*n* = 8) and HCT116 cells (*n* = 8). * denotes significant difference relative to adult cardiac myocyte (*P* < 0.05). (ii) Fluorescent staining of mitochondria (with MitoTracker Red), cytoplasm (with calcein), and nuclei (with Hoechst 33342) in adult or neonatal rat ventricular myocytes (excitation 555, 488, and 405 nm, respectively; emission collected at 580 ± 20, 520 ± 20, and 450 ± 20 nm). Mitochondrial distribution is more ordered and denser in adult myocytes compared with neonatal cells. (iii) Quantifying non-mitochondrial cell area. The mean intensity of MitoTracker Red fluorescence (*F*_mean_) was measured in calcein-positive Hoechst 33342-negative pixels (i.e. non-nuclear cytoplasm). Non-nuclear cytoplasmic pixels with MitoTracker Red signals <0.5 × *F*_mean_ were defined as belonging to non-mitochondrial regions of the myocyte. The percentage of these pixels (relative to all non-nuclear cytoplasmic pixels) is lower in adult (*n* = 9/3) myocytes compared with neonatal (*n* = 12/3) myocytes, consistent with faster calcein diffusion in the latter. (*C*) Comparison of *D*_cAMP_ (measured using dual microperfusion technique) with cytoplasmic diffusivity of the following fluorescent molecules (measured by FRAP): fluorescein (*n* = 8/3), MagFluo4 (*n* = 8/3), cAMP-fluorescein (*n* = 13/3) in control adult myocytes, cAMP-fluorescein (*n* = 12/3) in 5 µM 6-Bnz-cAMP-loaded adult myocytes, and cAMP-fluorescein diffusivity in neonatal myocytes (*n* = 6/4). Symbol *α* denotes significant difference (*P* < 0.05) relative to adult myocytes. (*D*) cAMP-fluorescein diffusivity (from C) and normalized calcein diffusivity (from *B*i) plotted as a function of non-mitochondrial area fraction of myocyte (*B*iii). Star denotes diffusivity in water. Dashed line plots equation for diffusivity in tortuous medium, normalized to diffusivity in water: *D*/*D*_0_ = (2−2 × *θ*)/(2 + *θ*), where *θ* is the effective volume fraction of spherical impermeable inclusions.

*Figure 6A*i summarizes the data for *D*_cAMP_ obtained under a range of conditions. Similar values were obtained in all types of experiment. There was no significant correlation between mean [cAMP] attained during the establishment of gradients and measured *D*_cAMP_ (*Figure 6A*ii; Pearson correlation coefficient: *R*^2^ = 0.06; ANOVA *F*= 0.31, *P*= 0.91). *D*_cAMP_ pooled for all experiments using ISO was 32.3 ± 7.6 µm^2^/s, which is an order of magnitude slower than in pure water.

### 3.10 Measuring cytoplasmic tortuosity to diffusion

Low *D*_cAMP_ may be explained by physical barriers imposed by macromolecules (e.g. proteins) and organelles (e.g. mitochondria), collectively called tortuosity. Cytoplasmic tortuosity to diffusion was probed using calcein, a fluorescent dye that can be loaded rapidly (as the AM-ester for <3 min) into the cytoplasm with minimal partitioning into subcellular organelles. Once in cytoplasm, calcein is not buffered and does not react chemically (processes that would otherwise restrict its spread). Cytoplasmic calcein diffusivity was measured using a FRAP protocol (see *Figure 2A*) and normalized to its diffusivity in water at 37°C (average: 604 µm^2^/s; range 330–1043 µm^2^/s).^35–37^ For rod-shaped adult myocytes, a one-dimensional algorithm was used to analyse data (as used in *Figure 2A*). For neonatal ventricular myocytes and two epithelial cell lines (HEK293 and HCT116), analysis used a two-dimensional solver to account for their non-rectangular shape. Physical barriers present in adult ventricular myocytes reduced calcein diffusivity by an order of magnitude (*Figure 6B*i). Tortuosity in adult myocytes was greater than in neonatal myocytes or in the two epithelial cell lines (*Figure 6B*i). Hypotonic swelling of adult myocytes (achieved by reducing superfusate osmolarity from 300 to 200 mOsm/kg; see Supplementary material online, *Figure S5A*) to dilute macromolecules did not significantly affect tortuosity (*Figure 6B*i). Disrupting T-tubules^38^ (detubulation; see Supplementary material online, *Figure S5B*) had only a minor effect on tortuosity (*Figure 6B*i). Mitochondria are, collectively, the most abundant organelle in adult myocytes (32% of cell volume, cf. cytoplasm occupies 7% of cell volume)^39^ and therefore a substantial physical barrier to the movement of charged molecules in cytoplasm.^40^ Mitochondrial density and order, quantified in terms of the distribution of MitoTracker Red fluorescence in confocally acquired images, were lower in neonatal myocytes compared with adult cells (*Figure 6B*ii). The non-mitochondrial area of a myocyte was quantified by measuring the fraction of non-nuclear cytoplasmic pixels (calcein-positive, Hoechst-negative) that have a MitoTracker Red signal less than half of the cell-averaged signal (*Figure 6B*iii). By this measure, neonatal myocytes had three-fold more non-mitochondrial space compared with adults, in good agreement with less restricted calcein diffusivity in the former.

### 3.11 Cytoplasmic cAMP diffusivity is similar to that of similar sized molecules

To compare the diffusivity of cAMP (mol. wt 329 g/mol) with that of similar sized molecules, the FRAP protocol was performed on myocytes loaded with fluorescent markers. Fluorescein (free acid; mol. wt 376 g/mol) was permeated into cells for 3 min in an acidic (pH 6.6) loading solution for improved cellular uptake (this loading protocol minimizes the degree of mitochondrial sequestration of dye compared with loading of the diacetate-ester). Another dye, MagFluo4 (AM-ester; mol. wt 525 g/mol) was loaded for 2 min (this dye shows minimal partitioning into mitochondria). Fluorescence images of myocytes loaded with fluorescein or MagFluo4 (superfused in Na^+^-free, Ca^2+^-free solution to block contraction artefacts) demonstrated no evidence for subcellular compartmentalization (see Supplementary material online, *Figure S6*). Intracellular diffusivity (see Supplementary material online, *Figure S6*) of fluorescein and MagFluo4 was 37.0 ± 4.5 and 37.9 ± 4.4 µm^2^/s, respectively (*Figure 6C*; *P*= 0.89). These values are similar to *D*_cAMP_ and suggest that the diffusive restrictions imposed upon the cytoplasmic mobility of fluorescein, MagFluo4, and cAMP are equal and relate to a common, chemically non-selective physical barrier, consistent with tortuosity (cAMP vs. MagFluo4, *P*= 0.66; cAMP vs. Fluorescein, *P*= 0.73).

### 3.12 Diffusivity of fluorescein-conjugated cAMP is slower than endogenous cAMP, as expected from the difference in molecular weight

One approach to estimating the diffusion of a non-fluorescent molecule (e.g. cAMP) is to conjugate it with a fluorescent compound. Diffusivity of cAMP-fluorescein (8-[Fluorescein]-cAMP; mol. wt 816 g/mol) was measured in adult myocytes using the same approach as for fluorescein (see Supplementary material online, *Figure S6*). The conjugate diffused at 26.2 ± 1.8 µm^2^/s, i.e. slower than cAMP or fluorescein (*Figure 6C*; *P* = 0.025), which is expected from the difference in molecular weight. To test whether cAMP-fluorescein diffusivity is affected by cAMP buffering, measurements were performed on myocytes pre-loaded with the AM-ester of 6-Bnz-cAMP (5 µM); cAMP-fluorescein diffusivity (25.0 ± 2.2 µm^2^/s) was not significantly different from that obtained in the absence of 6-Bnz-cAMP (*Figure 6C*; *P* = 0.68). In summary, fluorescent conjugates of cAMP diffuse slower than endogenous cAMP, and their mobility is not restricted by cAMP buffering. cAMP-fluorescein diffusivity was also measured in neonatal myocytes. In agreement with a less tortuous environment for diffusion owing to lower mitochondrial density and order, the cAMP conjugate diffused 3.4-fold faster in neonatal myocytes compared with adult cells (*Figure 6C*; *P*< 10^−4^).

## 4. Discussion

### 4.1 A systems approach to solving *D*_cAMP_

This study provides a measurement of diffusivity, at 37°C, of endogenously produced cAMP in the cytoplasm of the adult mammalian cardiac ventricular myocyte, a cell type that engages in cAMP microdomain signalling. Using a combination of genetic, microfluidic, and computational techniques, *D*_cAMP_ was measured to be 32 µm^2^/s over the physiological [cAMP] range. The experimental protocol was designed to provide sufficient information to solve, on a cell-by-cell basis, the diffusion reaction equation that describes spatio-temporal [cAMP] dynamics (*Figure 1D*). By accounting for cAMP synthesis and degradation, the method can dissect the diffusive fluxes from the chemical reactions that collectively determine [cAMP]. This approach derives a true estimate of cytoplasmic cAMP diffusivity.

### 4.2 Evoking and measuring [cAMP] gradients

The methods used herein have superior power to resolve *D*_cAMP_, because they combine advances in genetically encoded sensors and microfluidics. (i) [cAMP] was measured with H187, a highly sensitive cAMP sensor with a wide dynamic range of fluorescence that can be calibrated in units of concentration. H187 cannot facilitate cAMP diffusion, because the sensor's diffusivity is 15 times lower than *D*_cAMP_ (*Figure 2A*). Buffering by H187 is not sufficient to meaningfully reduce cAMP diffusion, as shown by the similarity in the longitudinal [cAMP] profiles reported by H187 in transduced cells and SERCA activity (a cAMP bioassay) in wild-type cells (*Figure 2B*). (ii) A dual microperfusion system, which releases two sharply separated microstreams with minimal mixing,^25,26^ was used to deliver a constant concentration of agonist to one half of the myocyte and antagonist to the other. This evoked a localized net production of cAMP in a controlled, reproducible, and robust manner. Diffusive coupling between the net cAMP-producing and net cAMP-consuming halves of the cell generates a smooth longitudinal [cAMP] profile that approaches a steady state and therefore can be time-averaged to improve signal-to-noise ratio. Deriving *D*_cAMP_ near the steady state allows fast and slow buffers, if present, to exert any restrictive effect on cAMP diffusion (diffusivity under out-of-equilibrium buffering tends to be faster than at the steady state, as shown for Ca^2+^ ions).^13,41^ This approach optimizes the conditions for revealing any meaningful effect of buffers on cAMP.

### 4.3 Cytoplasmic cAMP diffusivity is not reduced by buffering

cAMP binding to proteins, such as PKA, can in principle restrict cAMP mobility, but several lines of evidence suggest that this is not a major factor in determining *D*_cAMP_. Firstly, buffering would normally result in a positive relationship between [cAMP] and *D*_cAMP_, because raising ligand concentration would saturate buffer sites, placing a limit on their ability to restrict ligand diffusion. However, such a relationship was not observed over a broad concentration range tested up to 9 µM, a concentration that would saturate PKA sites (0.5–1 µM,^6^ *Figure 6A*ii). Secondly, buffer-restricted diffusion would be reversed by saturating buffering sites with a ligand-analogue. However, loading cytoplasm with the analogue 6-Bnz-cAMP did not raise *D*_cAMP_ (*Figure 5*). Similarly, 6-Bnz-cAMP did not accelerate the diffusivity of a fluorescently labelled cAMP derivative (*Figure 6C*). Also, manipulating cytoplasmic pH did not affect *D*_cAMP_, indicating that the protonation state of cAMP or its potential binding proteins does not affect mobility (*Figure 4*). Overall, these results indicate that low cAMP diffusivity cannot be explained by buffering alone.

### 4.4 Tortuosity is a principal reason for low cytoplasmic cAMP diffusivity

Cytoplasmic *D*_cAMP_ is an order of magnitude lower than in aqueous solution, a difference that cannot be explained by buffering. Molecules of similar size to cAMP but of different chemical identity, such as Magfluo4 and fluorescein, diffuse at a comparable rate to *D*_cAMP_. This argues for a common underlying cause of restricted movement. Previous studies have demonstrated that physical barriers present inside muscle cells halve the mobility of small ions,^42^ but larger molecules generally experience a greater restriction to movement.^43^ The degree to which physical barriers, such as proteins, organelles, or surface membrane invaginations (all of which are present in adult ventricular myocytes^28,42^), restrict cytoplasmic diffusion was probed using calcein, an inert and unbuffered cytoplasmic marker (*Figure 6B*). These measurements show that physical barriers collectively produce a tortuous environment that reduces calcein diffusivity by an order of magnitude. Tortuosity does not arise from macromolecules or intact T-tubules, because hypotonic swelling (which dilutes macromolecules) and detubulation did not greatly affect diffusivity (*Figure 6B*). Instead, tortuosity was related to mitochondrial order and density: the more disordered and lower density of mitochondria in neonatal myocytes allowed for faster cytoplasmic diffusion of calcein (*Figure 6B*) and a fluorescent cAMP derivative (*Figure 6C*). The importance of mitochondrial density in influencing cytoplasmic diffusion is illustrated in *Figure 6D*. cAMP-fluorescein and calcein diffused faster in myocytes with a greater non-mitochondrial area fraction, and both followed a similar relationship^44^ (since cAMP-fluorescein diffuses 20% slower than cAMP, the expected cAMP-fluorescein diffusivity in pure water is 80% of 444 = 360 µm^2^/s). A recent study^40^ has proposed that tortuosity in adult cardiac myocytes is explained in terms of permeability barriers situated every 1 µm in the transverse and longitudinal directions, equivalent to the periodicity of mitochondria. This adds to the evidence that a principal contributor to the tortuosity in adult myocytes is the highly ordered and dense array of mitochondria.

The importance of mitochondria in restricting the diffusivity of a membrane-permeant fluorescent cAMP analogue was recently reported by Agarwal and colleagues,^19^ but the effect was attributed to buffering by PKA moieties at the outer mitochondrial membrane (OMM). Lipophilicity of the cAMP analogue may underestimate the effect of physical barriers imposed by mitochondrial membranes, which could explain why tortuosity was not considered. However, even in experiments that dislodge fixed buffer sites from the OMM, diffusivity of the cAMP analogue was much slower than in water; thus, the importance of tortuosity cannot be excluded. Furthermore, diffusivity of the cAMP analogue did not change in the presence of IBMX, a drug that is expected to raise [cAMP] and reduce the number of PKA moieties available for buffering and restricting cAMP movement. In contrast, our measurements of endogenously produced cAMP provide three lines of evidence against a dominant role of buffering in restricting diffusion. Further studies are warranted to quantify the importance of buffering relative to tortuosity in restricting cAMP diffusion at different spatial scales in the cardiac myocyte.

### 4.5 Importance of tortuosity for cAMP microdomain signalling

At the subcellular scale, myocyte tortuosity is highly heterogeneous, and discrete barriers to diffusion are plausible boundaries for cAMP microdomains.^6^ [cAMP] measurements with sub-sarcomeric resolution are not yet available to map the source of tortuosity, but recent findings^40^ have proposed that diffusion barriers are situated at Z and M lines. Our measurement of global *D*_cAMP_ can be used to estimate the permeability of these barriers. As explained in the Supplementary material online (equations), 92% of overall resistance to cAMP movement would occur at these barriers, and a stochastic model of diffusion^40^ predicts that ∼1 in 2000 cAMP molecules would cross such a barrier. This small leakage of cAMP across barriers would be readily degraded by PDE activity concentrated near Z and M lines.^45^ The cAMP molecules reflected off the barrier would contribute towards the formation of a concentrated sub-sarcomeric cAMP microdomain. In contrast, earlier estimates of *D*_cAMP_ (200–500 µm^2^/s)^18,20,21^ would imply a much more permeable barrier, allowing a leak of >1 in 20 molecules. Indeed, several computational models^6,17,18^ have deemed these faster diffusivities to be incompatible with local cAMP signalling, because the enzymatic capacity of native PDE to degrade cAMP would be insufficient to fully restrict a locally triggered cAMP signal. Since our estimate of *D*_cAMP_ is ∼10 times lower than the average of previously reported diffusivities, Eq. (1) predicts a 10-fold greater peak [cAMP] amplitude near the source (e.g. adenylyl cyclase). This would result in a more potent activation of target proteins locally. Tortuosity is highly cell type dependent; therefore, differences in cAMP signalling may relate to differences in *D*_cAMP_. On the basis that cAMP-fluorescein diffusivity in neonatal myocytes was 3.4-fold faster than in adult myocytes (*Figure 6A*), neonatal myocyte *D*_cAMP_ is estimated to be 110 µm^2^/s. This difference in *D*_cAMP_ should be considered when comparing cAMP signalling in neonatal and adult myocytes.

### 4.6 Conclusions

We have measured the cAMP diffusion coefficient in cardiac myocyte cytoplasm to be 32 µm^2^/s. The lack of a clear [cAMP] dependence of *D*_cAMP_ and the absence of an accelerating effect of saturating cAMP-binding sites with a cAMP analogue argue against a meaningful effect of buffering on diffusivity. Physical restrictions imposed by cardiac myocyte ultrastructure are sufficient to explain why *D*_cAMP_ in cytoplasm is considerably lower than in pure water. The present estimate of *D*_cAMP_ in adult myocytes is substantially lower than earlier measurements made in cardiac and non-cardiac tissue (range: 130–500 µm^2^/s).^15,18,20–22^ Considering that the lattice structure of the adult ventricular myocyte imposes barriers every ∼1 µm, our measurement of *D*_cAMP_ is more compatible with the formation of concentrated cAMP microdomains at the sub-sarcomeric level. Faster cAMP diffusivity in neonatal myocytes argues for a different morphology of microdomain signalling, which may relate to changes in cAMP signalling at distinct stages of development. It is possible that aberrant forms of cAMP signalling may relate to changes in tortuosity in myocytes from diseased hearts. It is well established that myocytes from failing hearts have altered mitochondrial morphology^46–48^, which may affect *D*_cAMP_ and could contribute towards aberrant cAMP microdomain signalling. This work emphasizes the importance of cytoplasmic tortuosity in shaping cardiac cAMP microdomains and highlights the need to study cAMP signalling in its relevant cellular context.

## Supplementary material

Supplementary material is available at *Cardiovascular Research* online.

## Funding

This work was supported by the British Heart Foundation (P.S., grant PG/12/2/29324), Royal Society University Research Fellowship (P.S.), British Heart Foundation Centre of Research Excellence Fellowship (K.L., RE/13/1/30181), and Wellcome Trust Studentship (O.L.). Funding to pay the Open Access publication charges for this article was provided by …

## References

[1] Lomas O, Zaccolo M Phosphodiesterases maintain signaling fidelity via compartmentalization of cyclic nucleotides. *Physiology (Bethesda)* 2014;29:141–149. 24583770 10.1152/physiol.00040.2013 PMC3949206

[2] Lefkimmiatis K, Zaccolo M cAMP signaling in subcellular compartments. *Pharmacol Ther* 2014;143:295–304. 24704321 10.1016/j.pharmthera.2014.03.008 PMC4117810

[3] Skalhegg BS, Tasken K Specificity in the cAMP/PKA signaling pathway. Differential expression,regulation, and subcellular localization of subunits of PKA. *Front Biosci* 2000;5:D678–D693. 10922298 10.2741/skalhegg

[4] Smith FD, Scott JD A-kinase-anchoring protein-Lbc connects stress signaling to cardiac hypertrophy. *Mol Cell Biol* 2013;33:2–3. 23149943 10.1128/MCB.01490-12 PMC3536311

[5] Kritzer MD, Li J, Dodge-Kafka K, Kapiloff MS AKAPs: the architectural underpinnings of local cAMP signaling. *J Mol Cell Cardiol* 2012;52:351–358. 21600214 10.1016/j.yjmcc.2011.05.002 PMC3168680

[6] Saucerman JJ, Greenwald EC, Polanowska-Grabowska R Mechanisms of cyclic AMP compartmentation revealed by computational models. *J Gen Physiol* 2014;143:39–48. 24378906 10.1085/jgp.201311044 PMC3874575

[7] Steinberg SF, Brunton LL Compartmentation of G protein-coupled signaling pathways in cardiac myocytes. *Annu Rev Pharmacol Toxicol* 2001;41:751–773. 11264475 10.1146/annurev.pharmtox.41.1.751

[8] Fischmeister R, Castro LR, Abi-Gerges A, Rochais F, Jurevicius J, Leroy J, Vandecasteele G Compartmentation of cyclic nucleotide signaling in the heart: the role of cyclic nucleotide phosphodiesterases. *Circ Res* 2006;99:816–828. 17038651 10.1161/01.RES.0000246118.98832.04

[9] Zaccolo M, Pozzan T Discrete microdomains with high concentration of cAMP in stimulated rat neonatal cardiac myocytes. *Science* 2002;295:1711–1715. 11872839 10.1126/science.1069982

[10] Scott JD, Dessauer CW, Tasken K Creating order from chaos: cellular regulation by kinase anchoring. *Annu Rev Pharmacol Toxicol* 2013;53:187–210. 23043438 10.1146/annurev-pharmtox-011112-140204 PMC3540170

[11] Zaccolo M Spatial control of cAMP signalling in health and disease. *Curr Opin Pharmacol* 2011;11:649–655. 22000603 10.1016/j.coph.2011.09.014 PMC4126235

[12] Otero C, Penaloza JP, Rodas PI, Fernandez-Ramires R, Velasquez L, Jung JE Temporal and spatial regulation of cAMP signaling in disease: role of cyclic nucleotide phosphodiesterases. *Fundam Clin Pharmacol* 2014;28:593–607. 24750474 10.1111/fcp.12080

[13] Neher E Usefulness and limitations of linear approximations to the understanding of Ca++ signals. *Cell Calcium* 1998;24:345–357. 10091004 10.1016/s0143-4160(98)90058-6

[14] Kar P, Parekh AB Distinct spatial Ca2+ signatures selectively activate different NFAT transcription factor isoforms. *Mol Cell* 2015;58:232–243. 25818645 10.1016/j.molcel.2015.02.027 PMC4405353

[15] Nikolaev VO, Bunemann M, Schmitteckert E, Lohse MJ, Engelhardt S Cyclic AMP imaging in adult cardiac myocytes reveals far-reaching beta1-adrenergic but locally confined beta2-adrenergic receptor-mediated signaling. *Circ Res* 2006;99:1084–1091. 17038640 10.1161/01.RES.0000250046.69918.d5

[16] Dworkin M, Keller KH Solubility and diffusion coefficient of adenosine 3′:5′-monophosphate. *J Biol Chem* 1977;252:864–865. 14137

[17] Iancu RV, Jones SW, Harvey RD Compartmentation of cAMP signaling in cardiac myocytes: a computational study. *Biophys J* 2007;92:3317–3331. 17293406 10.1529/biophysj.106.095356 PMC1852367

[18] Saucerman JJ, Zhang J, Martin JC, Peng LX, Stenbit AE, Tsien RY, McCulloch AD Systems analysis of PKA-mediated phosphorylation gradients in live cardiac myocytes. *Proc Natl Acad Sci USA* 2006;103:12923–12928. 16905651 10.1073/pnas.0600137103 PMC1568947

[19] Agarwal SR, Clancy CE, Harvey RD Mechanisms restricting diffusion of intracellular cAMP. *Sci Rep* 2016;6:19577. 26795432 10.1038/srep19577 PMC4726171

[20] Chen C, Nakamura T, Koutalos Y Cyclic AMP diffusion coefficient in frog olfactory cilia. *Biophys J* 1999;76:2861–2867. 10233102 10.1016/S0006-3495(99)77440-0 PMC1300257

[21] Huang RC, Gillette R Kinetic analysis of cAMP-activated Na+ current in the molluscan neuron. A diffusion-reaction model. *J Gen Physiol* 1991;98:835–848. 1720449 10.1085/jgp.98.4.835 PMC2229076

[22] Nikolaev VO, Bunemann M, Hein L, Hannawacker A, Lohse MJ Novel single chain cAMP sensors for receptor-induced signal propagation. *J Biol Chem* 2004;279:37215–37218. 15231839 10.1074/jbc.C400302200

[23] Filadi R, Pozzan T Generation and functions of second messengers microdomains. *Cell Calcium* 2015;58:405–414. 25861743 10.1016/j.ceca.2015.03.007

[24] Lomas O, Brescia M, Carnicer R, Monterisi S, Surdo NC, Zaccolo M Adenoviral transduction of FRET-based biosensors for cAMP in primary adult mouse cardiomyocytes. *Methods Mol Biol* 2015;1294:103–115. 25783880 10.1007/978-1-4939-2537-7_8

[25] Swietach P, Youm JB, Saegusa N, Leem CH, Spitzer KW, Vaughan-Jones RD Coupled Ca2+/H+ transport by cytoplasmic buffers regulates local Ca2+ and H+ ion signaling. *Proc Natl Acad Sci USA* 2013;110:E2064–E2073. 23676270 10.1073/pnas.1222433110 PMC3670334

[26] Swietach P, Leem CH, Spitzer KW, Vaughan-Jones RD Experimental generation and computational modeling of intracellular pH gradients in cardiac myocytes. *Biophys J* 2005;88:3018–3037. 15653720 10.1529/biophysj.104.051391 PMC1305395

[27] Jurevicius J, Fischmeister R cAMP compartmentation is responsible for a local activation of cardiac Ca2+ channels by beta-adrenergic agonists. *Proc Natl Acad Sci USA* 1996;93:295–299. 8552625 10.1073/pnas.93.1.295 PMC40225

[28] Parfenov AS, Salnikov V, Lederer WJ, Lukyanenko V Aqueous diffusion pathways as a part of the ventricular cell ultrastructure. *Biophys J* 2006;90:1107–1119. 16284268 10.1529/biophysj.105.071787 PMC1367097

[29] Tian Q, Pahlavan S, Oleinikow K, Jung J, Ruppenthal S, Scholz A, Schumann C, Kraegeloh A, Oberhofer M, Lipp P, Kaestner L Functional and morphological preservation of adult ventricular myocytes in culture by sub-micromolar cytochalasin D supplement. *J Mol Cell Cardiol* 2012;52:113–124. 21930133 10.1016/j.yjmcc.2011.09.001

[30] Klarenbeek J, Goedhart J, van Batenburg A, Groenewald D, Jalink K Fourth-generation epac-based FRET sensors for cAMP feature exceptional brightness, photostability and dynamic range: characterization of dedicated sensors for FLIM, for ratiometry and with high affinity. *PLoS One* 2015;10:e0122513. 25875503 10.1371/journal.pone.0122513 PMC4397040

[31] Hulikova A, Swietach P Rapid CO2 permeation across biological membranes: implications for CO2 venting from tissue. *FASEB J* 2014;28:2762–2774. 24652949 10.1096/fj.13-241752

[32] Lefkimmiatis K, Moyer MP, Curci S, Hofer AM ‘cAMP sponge’: a buffer for cyclic adenosine 3′, 5’-monophosphate. *PLoS One* 2009;4:e7649. 19888343 10.1371/journal.pone.0007649 PMC2766031

[33] Feinstein WP, Zhu B, Leavesley SJ, Sayner SL, Rich TC Assessment of cellular mechanisms contributing to cAMP compartmentalization in pulmonary microvascular endothelial cells. *Am J Physiol Cell Physiol* 2012;302:C839–C852. 22116306 10.1152/ajpcell.00361.2011 PMC3311237

[34] Christensen AE, Selheim F, de Rooij J, Dremier S, Schwede F, Dao KK, Martinez A, Maenhaut C, Bos JL, Genieser HG, Doskeland SO cAMP analog mapping of Epac1 and cAMP kinase. Discriminating analogs demonstrate that Epac and cAMP kinase act synergistically to promote PC-12 cell neurite extension. *J Biol Chem* 2003;278:35394–35402. 12819211 10.1074/jbc.M302179200

[35] Papadopoulos MC, Kim JK, Verkman AS Extracellular space diffusion in central nervous system: anisotropic diffusion measured by elliptical surface photobleaching. *Biophys J* 2005;89:3660–3668. 16143636 10.1529/biophysj.105.068114 PMC1366858

[36] Yoshida N, Tamura M, Kinjo M Fluorescence correlation spectroscopy: a new tool for probing the microenvironment of the internal space of organelles. *Single Mol* 2000;1:279.

[37] Becker S, Zorec B, Miklavcic D, Pavselj N Transdermal transport pathway creation: electroporation pulse order. *Math Biosci* 2014;257:60–68. 25017876 10.1016/j.mbs.2014.07.001

[38] Kawai M, Hussain M, Orchard CH Excitation-contraction coupling in rat ventricular myocytes after formamide-induced detubulation. *Am J Physiol* 1999;277:H603–H609. 10444485 10.1152/ajpheart.1999.277.2.H603

[39] Barth E, Stammler G, Speiser B, Schaper J Ultrastructural quantitation of mitochondria and myofilaments in cardiac muscle from 10 different animal species including man. *J Mol Cell Cardiol* 1992;24:669–681. 1404407 10.1016/0022-2828(92)93381-s

[40] Illaste A, Laasmaa M, Peterson P, Vendelin M Analysis of molecular movement reveals latticelike obstructions to diffusion in heart muscle cells. *Biophys J* 2012;102:739–748. 22385844 10.1016/j.bpj.2012.01.012 PMC3283817

[41] Smith GD, Keizer JE, Stern MD, Lederer WJ, Cheng H A simple numerical model of calcium spark formation and detection in cardiac myocytes. *Biophys J* 1998;75:15–32. 9649364 10.1016/S0006-3495(98)77491-0 PMC1299676

[42] Kushmerick MJ, Podolsky RJ Ionic mobility in muscle cells. *Science* 1969;166:1297–1298. 5350329 10.1126/science.166.3910.1297

[43] Mastro AM, Babich MA, Taylor WD, Keith AD Diffusion of a small molecule in the cytoplasm of mammalian cells. *Proc Natl Acad Sci USA* 1984;81:3414–3418. 6328515 10.1073/pnas.81.11.3414 PMC345518

[44] Nitsche JM Cellular microtransport processes: intercellular, intracellular, and aggregate behavior. *Annu Rev Biomed Eng* 1999;1:463–503. 11701497 10.1146/annurev.bioeng.1.1.463

[45] Mongillo M, McSorley T, Evellin S, Sood A, Lissandron V, Terrin A, Huston E, Hannawacker A, Lohse MJ, Pozzan T, Houslay MD, Zaccolo M Fluorescence resonance energy transfer-based analysis of cAMP dynamics in live neonatal rat cardiac myocytes reveals distinct functions of compartmentalized phosphodiesterases. *Circ Res* 2004;95:67–75. 15178638 10.1161/01.RES.0000134629.84732.11

[46] Dorn GW 2nd, Kitsis RN The mitochondrial dynamism-mitophagy-cell death interactome: multiple roles performed by members of a mitochondrial molecular ensemble. *Circ Res* 2015;116:167–182. 25323859 10.1161/CIRCRESAHA.116.303554 PMC4282600

[47] Ong SB, Hausenloy DJ Mitochondrial morphology and cardiovascular disease. *Cardiovasc Res* 2010;88:16–29. 20631158 10.1093/cvr/cvq237 PMC2936127

[48] Rosca MG, Tandler B, Hoppel CL Mitochondria in cardiac hypertrophy and heart failure. *J Mol Cell Cardiol* 2013;55:31–41. 22982369 10.1016/j.yjmcc.2012.09.002 PMC3805050

